# Salivary Hyalinizing Clear Cell Carcinoma and Odontogenic Clear Cell Carcinoma: A Case Series and a Scoping Review Comparing Clinicopathological Presentations

**DOI:** 10.3390/diagnostics16121846

**Published:** 2026-06-15

**Authors:** Primali Rukmal Jayasooriya, Sumedha Madhavie Range, Ayodya Methmini Fernando, Balapuwaduge Ranjit Rigobert Nihal Mendis, Tommaso Lombardi

**Affiliations:** 1 Department of Oral Pathology, Faculty of Dental Sciences, University of Peradeniya, Peradeniya 20400, Sri Lanka; primalijaya@dental.pdn.ac.lk (P.R.J.); d03497@pgim.cmb.ac.lk (S.M.R.); ayodyamethmini99@gmail.com (A.M.F.); ranjitrigobert@gmail.com (B.R.R.N.M.); 2 Postgraduate Institute of Medicine, No 160, Nandadasa Kodagoda Mawatha, Colombo 00700, Sri Lanka; 3 Unit of Oral Medicine and Oral Maxillofacial Pathology, Division of Oral and Maxillofacial Surgery, Department of Surgery, Faculty of Medicine, University Hospitals of Geneva, University of Geneva, 1205 Geneva, Switzerland

**Keywords:** hyalinizing clear cell carcinoma, clear cell odontogenic carcinoma, minor and major salivary glands, gnathic bones, maxilla, palate, odontogenic tumour, salivary tumour

## Abstract

**Background/Objectives:** Hyalinizing clear cell carcinoma (HCCC) and clear cell odontogenic carcinoma (CCOC) are rare clear cell neoplasms with overlapping histopathological features. This study aimed to compare their clinicopathological characteristics, particularly in anatomically challenging sites such as the palate and maxilla. **Methods:** Three analyses were performed. First, an unpublished series of five HCCC and three CCOC cases was evaluated for diagnostic histopathological features. Second, a PRISMA-ScR-guided literature review of 58 HCCCs and 45 CCOCs restricted to tumours arising in intraoral minor salivary glands, major salivary glands and gnathic bones published between 2000 and 2025 was conducted using PubMed. Third, a sub-analysis compared palatal HCCC and maxillary CCOC (25 vs. 14 cases), integrating literature and unpublished cases. **Results:** The case series and overall literature review showed that HCCC and CCOC predominantly occurred in adults (mean age, case series: 50.8 years; literature: 56.33 years for HCCC and 61 vs. 54.11 years for CCOC) with a female predilection (case series: 60%; literature: 68%) and generally exhibited clinically indolent behaviour. The site of occurrence, soft tissue (HCCC) versus intraosseous location (CCOC), was the principal distinguishing feature. No marked differences were observed between the two tumours in either the overall literature analysis or the site-specific sub-analysis. However, CCOC at maxillary/palatal sites presented with a higher number of larger lesions and higher number of cases with nodal metastasis compared with HCCC, most probably indicating delayed clinical detection rather than intrinsic aggressiveness of CCOC. Histopathological overlap was considerable; however, diffuse dense hyalinization (4/5), focal glandular differentiation (2/5), mucous-secreting cells (4/5) and salivary gland association (5/5) favoured HCCC, whereas patchy hyalinization (3/3), larger tumour lobules (3/3) and peripheral palisading (2/3) favoured CCOC. **Conclusions:** HCCC and CCOC demonstrate clinicopathological similarities and shared EWSR1 rearrangement, supporting a close biological relationship. The considerable overlap between these tumours support the hypothesis that CCOC may represent the intraosseous counterpart of HCCC and highlight the importance of integrated clinicopathological assessment and further clarification in future WHO classifications.

## 1. Introduction

The 5th Edition (2022) of the World Health Organization Classification of Head and Neck Tumours recognizes clear cell morphology as a diagnostic feature shared by several distinct epithelial malignancies arising in the oral and maxillofacial region [1]. Among these, hyalinizing clear cell carcinoma (HCCC) of salivary origin and clear cell odontogenic carcinoma (CCOC) of gnathic bones are rare but clinically significant malignant entities with overlapping clinicopathological characteristics that may complicate diagnosis.

Elaborating further, HCCC is defined as a salivary gland malignancy composed of clear and eosinophilic cells in a variably hyalinized stroma, usually associated with EWSR1 rearrangement [1,2,3,4,5,6,7,8,9]. In contrast, CCOC is an odontogenic carcinoma characterized by sheets, cords and nests of clear cells in a fibro-cellular or hyalinized stroma, and it likewise harbours the EWSR1 translocation [1,9,10,11,12,13,14,15,16]. Thus, both tumours share notable morphological and genetic similarities, creating a potential diagnostic challenge [1,9,14,16]. This challenge is further amplified when HCCC arises in the palate and CCOC involves the maxilla, as their close anatomical relationship may obscure the tumour origin. HCCC is generally associated with a relatively indolent clinical course with low rates of locoregional recurrence and rare disease-related mortality except in cases of high-grade transformation [1,5]. Though CCOC has been also reported to have low-grade behaviour, higher rates of recurrence [1,11] and disease-related mortality [1] has been observed when compared with HCCC. Therefore, it is important to differentiate the two entities, with emphasis on awareness of their clinicopathological characteristics, although the World Health Organization Classification of Head and Neck Tumours suggests a common pathogenesis between the two tumours [1].

Failure to accurately distinguish between HCCC and CCOC may result in inappropriate treatment planning [17]. When an intraosseous clear cell malignancy is mistaken for a salivary gland tumour, or vice versa, patients may receive suboptimal treatment, including inadequate surgical margins, unnecessary or omitted adjuvant therapy and insufficient long-term follow-up, all of which may adversely influence outcomes. Therefore, the present study aimed to provide a structured comparative analysis of the two entities based on a case series diagnosed at the Department of Oral Pathology, Faculty of Dental Sciences, University of Peradeniya, together with cases identified through a literature review. By combining institutional experience with published evidence, this work seeks to improve diagnostic clarity and contribute meaningful regional data on these uncommon but clinically significant malignancies.

## 2. Materials and Methods

The clinicopathological study was designed as a retrospective analysis comprising a case series of HCCC and CCOC, along with a review of the literature. The case series component included 5 and 3 previously unpublished cases of HCCC and CCOC, respectively, retrieved from the archives of the Department of Oral Pathology, Faculty of Dental Sciences, University of Peradeniya, Peradeniya, Sri Lanka. Three CCOCs are unpublished in the context of the present topic, but two cases were included in a previous prevalence study from the same institution [18]. These were supplemented with additional published cases of 58 HCCCs [8,18,19,20,21,22,23,24,25,26,27,28,29,30,31,32,33,34,35,36,37,38,39,40,41,42,43,44,45,46,47,48,49,50,51,52,53,54,55,56,57] and 45 CCOCs [10,58,59,60,61,62,63,64,65,66,67,68,69,70,71,72,73,74,75,76,77,78,79,80,81,82,83,84] identified from the literature. The literature review component of the study was conducted in accordance with PRISMA-ScR principles.

### 2.1. Case Selection

After removing multiple biopsies from the same patients, 5 and 3 cases of HCCCs and CCOCs, respectively, diagnosed over a 20-year period from 2005 to 2025 were retrieved from the archives of the Department of Oral Pathology. Data on patient demographics, including age, sex, lesion site, size, duration and symptoms, as well as histopathology, management and recurrence, were obtained from an oral pathology database. Following confirmation of the histopathological diagnoses according to the 2022 WHO blue book on Head and Neck Tumours [1] by one of the authors (P.R.J.), cases were included in the study. H&E slides were reviewed to record tumour circumscription (encapsulated, unencapsulated and invasive or unencapsulated and well circumscribed), tumour cell components (clear cells, cells with eosinophilic cytoplasm and squamous differentiation), morphological patterns (large lobules, thin strands and individual cells), stromal hyalinization (mild, moderate or marked), perineural and vascular invasion (present/absent), necrosis (present/absent), mitotic activity (number per 10 HPF) for both tumours, high-grade differentiation for HCCC and peripheral palisading for CCOC. PAS- and DPAS-positive cells presenting with glycogen and mucin, respectively, were also recorded for both tumours.

### 2.2. Procedure of Literature Review

A comprehensive literature review was conducted to identify studies related to HCCC and CCOC. Electronic databases, including PubMed/MEDLINE, were systematically searched to include studies published from 2000 to December 2025 using the following search strings tailored to each tumour entity.

1. (“Clear Cell Carcinoma” [Title/Abstract] OR “Hyalinizing Clear Cell Carcinoma” [Title/Abstract] OR “Clear Cell Carcinoma of Salivary Gland” [Title/Abstract] OR “Salivary Gland Clear Cell Carcinoma” [Title/Abstract] OR “Hyalinizing Clear Cell Carcinoma of Salivary Gland” [Title/Abstract]OR “EWSR1-ATF1” [Title/Abstract] OR “EWSR1 rearrangement” [Title/Abstract]) AND (“Salivary Gland Neoplasms” [MeSH] OR “salivary gland tumor” [Title/Abstract] OR “minor salivary gland” [Title/Abstract])
2. (“Clear Cell Odontogenic Carcinoma” [Title/Abstract] OR “clear cell odontogenic tumor” [Title/Abstract] OR “clear cell odontogenic tumour” [Title/Abstract] OR “clear cell odontogenic neoplasm” [Title/Abstract] OR “odontogenic clear cell carcinoma” [Title/Abstract] OR “clear cell odontogenic carcinoma of jaw” [Title/Abstract] OR “CCOC” [Title/Abstract] OR “EWSR1-ATF1” [Title/Abstract] OR “EWSR1 rearrangement” [Title/Abstract]) AND (“Odontogenic Tumors” [MeSH] OR odontogenic [Title/Abstract] OR jaw [Title/Abstract] OR mandible [Title/Abstract] OR maxilla [Title/Abstract]).

**Figure 1:**
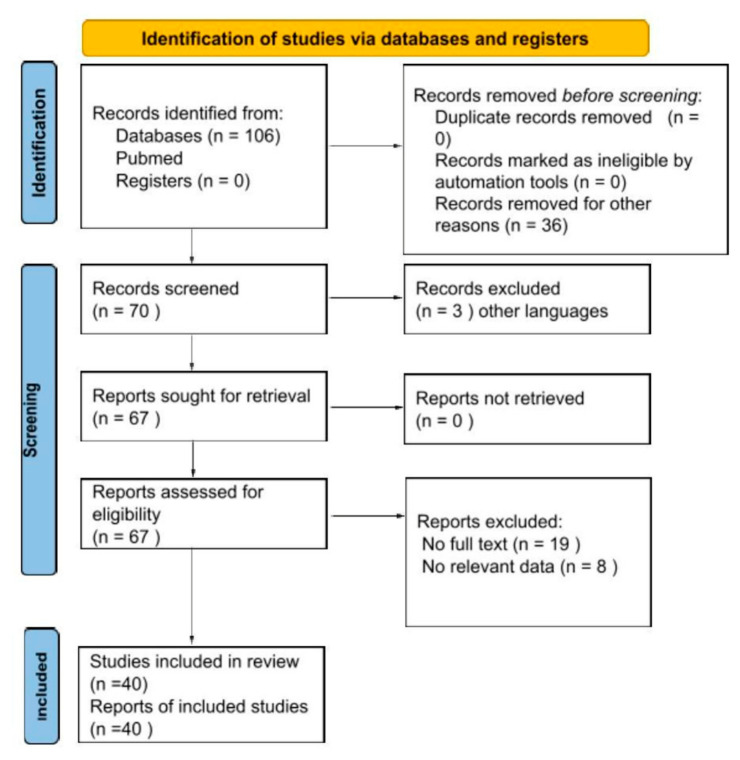
Flow chart illustrating the case selection process of HCCC.

**Figure 2:**
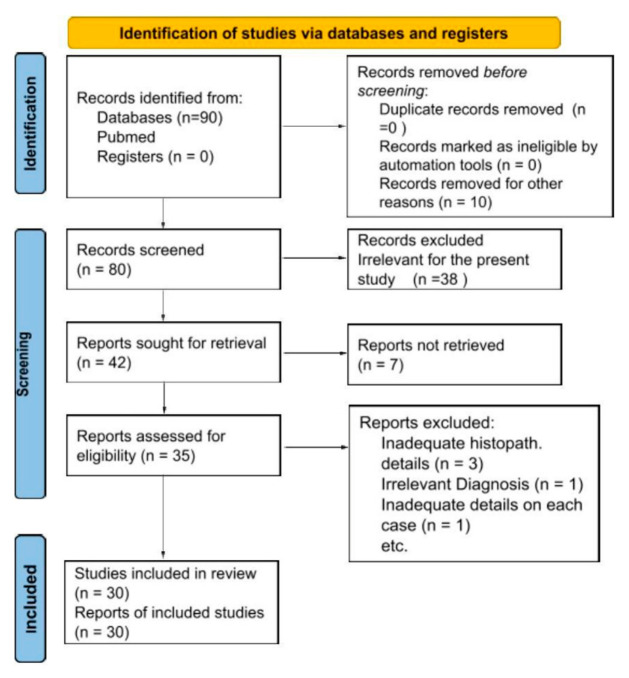
Flow chart illustrating the case selection process of CCOC.

Initially, duplicate entries were removed. Titles and abstracts were then independently screened by 2 reviewers for relevance, followed by full-text evaluation of potentially eligible studies based on the following inclusion and exclusion criteria (Figure 1 and Figure 2). Studies were included for the literature review component if they met the following criteria: population—human subjects diagnosed with HCCC or CCOC; diagnosis—confirmed by histopathological examination with or without supportive immunohistochemical or molecular findings; location—lesions arising from intraoral minor and major salivary glands pertaining to HCCC and from the jaw bones in regards to CCOC; study design—case series and case reports; and language—articles published in English. Studies were excluded if they were not peer-reviewed (e.g., letters to the editor or conference abstracts); were systematic reviews or meta-analyses, reports based on other clear cell tumours of salivary or odontogenic origin, focused on animal or in vitro studies; or lacked adequate clinical or histopathological data. Discrepancies between the two reviewers with respect to the selection process were resolved through consensus discussion.

From the eligible studies (40 HCCCs and 30 CCOCs), the following information was gathered manually, focusing on clinical features (age, gender, lesion size, location, and symptoms); histopathological findings (nodal metastasis, high grade differentiation); treatment approaches (surgical management with or without adjuvant therapy); and outcome (presence of recurrences, and follow-up outcomes) for the comparative analysis highlighting similarities and differences between HCCC and CCOC. Cases with a few incomplete clinicopathological information were retained where diagnosis was adequately confirmed, although missing variables were excluded from specific comparative analyses.

### 2.3. Comparative Analysis of Palatal HCCC and Maxillary CCOC

Thereafter, information gathered from the case series and literature review pertaining to palatal HCCC and maxillary CCOC were presented in isolation to identify any similarities or differences, as it is our experience that at these two locations, massive tumours that extend beyond the original site create significant diagnostic challenges.

## 3. Results

### 3.1. Case Series

With reference to Table 1A,B, the mean age at diagnosis for the five cases of HCCC was 50.8 years, with an age range of 22–58 years. A female predilection was observed, with a male-to-female ratio of 2:3. All tumours involved intraoral minor salivary glands, with three arising in the palatal minor salivary glands. Out of these three lesions, two showed extension into the maxilla; in one of the cases (HCCC case 2), the original palatal origin was identified based on a documented history of a palatal lesion. With respect to the size of the lesions, all except one were pT3 or pT4 lesions, with T4 lesions extending into surrounding bone/tissue. All tumours were managed surgically, and microscopically in all cases, the tumour was identified in at least one soft tissue/mucosal margin. Two patients presented with regional lymph node metastasis at diagnosis (HCCC case 3 and 5). Recurrences were detected in a single case (HCCC case 2) after 195 months of follow-up, while absent in two (HCCC case 1 and 5) out of three lesions with follow-up information.

The three cases of CCOC also occurred in adults, with a mean age of 61 years and an age range of 51–74 years. A female predilection was observed (male-to-female ratio of 1:2). Two lesions arose in the mandible, while one involved the maxilla. One patient developed metastatic disease two years following surgical treatment of a mandibular lesion (CCOC case 2). Another patient with a mandibular CCOC developed local recurrence with extension into the floor of the mouth and tongue 48 months after excision of the primary tumour (CCOC case 1).

**Figure 3:**
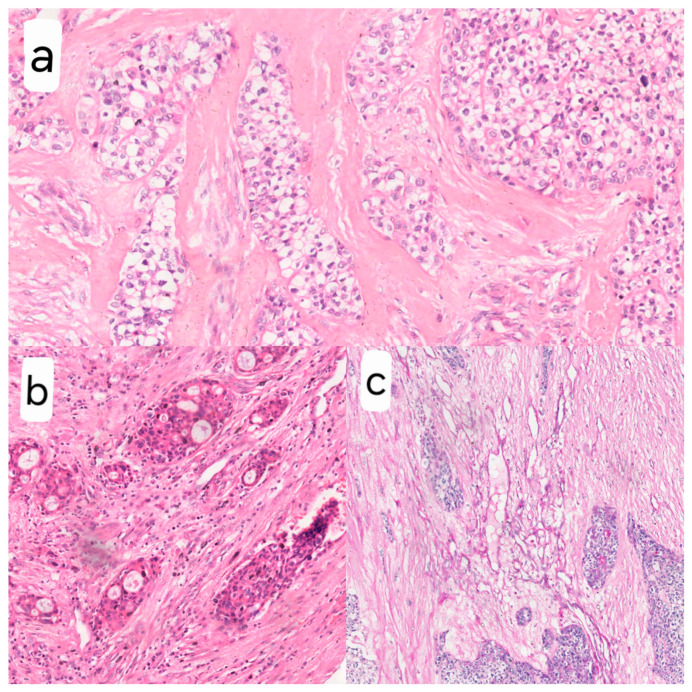
Images of HCCC case 1 showing an unencapsulated clear cell tumour composed of tumour lobules in a densely hyalinized stroma (**a**) and glandular differentiation (**b**) (H&E x20). (**c**) A DPAS-stained section reveals a few mucin-containing cells towards the five o’clock position of the image (DPAS x20).

Table 1B summarizes the histopathological findings of HCCC and CCOC. Accordingly, all HCCC cases were unencapsulated and demonstrated infiltrative growth, either invading adjacent skeletal muscle or merging with the surface oral epithelium. The tumours were composed of both clear and eosinophilic cells arranged in nests, strands and islands. In all tumours, clear cell component was the predominant cell type (Figure 3). A few mucous-secreting cells were identified in one case (HCCC case 1), while intracellular and extracellular mucin was demonstrated by diastase–periodic acid–Schiff (DPAS) staining in HCCC cases 1 and 3–5. Glandular differentiation was observed in HCCC case 1 and 2. With the exception of HCCC case 5, none of the tumours exhibited increased mitotic activity. The stroma was diffusely and densely hyalinized in four tumours (case 1, 2, 3, 4, 5) and focal in one (case 3). Further, all five lesions showed close proximity to salivary glands. Perineural and vascular invasion were identified in 40% (2/5) of cases.

**Figure 4:**
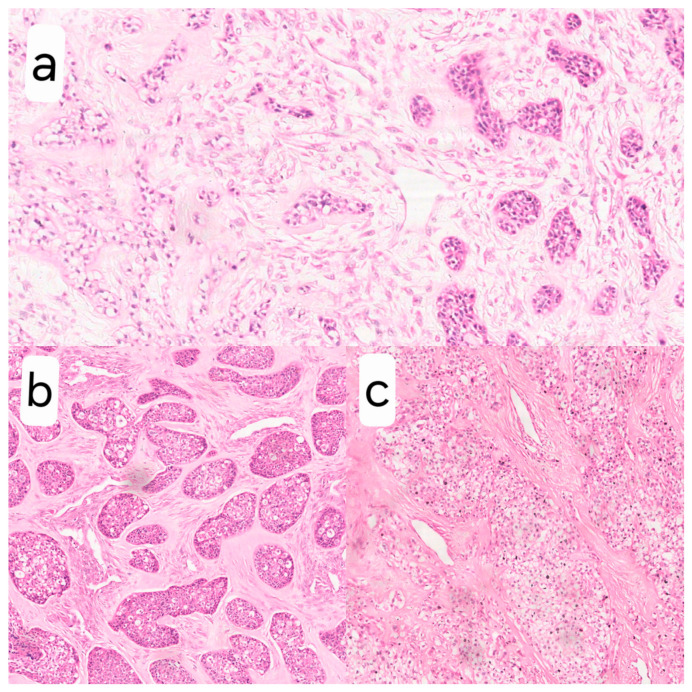
Images of CCOC case 1 showing an unencapsulated tumour composed of clear cells and cells with eosinophilic cytoplasm. Stromal hyalinization is more marked towards the area with the clear cell component while areas showing cells with eosinophilic cytoplasm show fibro-cellular stroma (**a**). Figures (**b**) and (**c**) show large tumour lobules composed of eosinophilic and clear cells, respectively (**b**,**c**) (H&E x20).

Histopathologically, all three cases of CCOCs were unencapsulated and composed of sheets and larger islands of relatively monomorphic tumour cells with clear and eosinophilic cytoplasms (Figure 4). Peripheral palisading with reverse nuclear polarity was observed focally in tumour islands in CCOC cases 1 and 3. Focal stromal hyalinization was present in all cases. CCOC case 2 exhibited a heterogeneous morphology, with areas composed of basaloid epithelial cells lacking cytoplasmic clearing. In all three cases, the clear cells were negative for mucin; however, they were periodic acid–Schiff (PAS)-positive and diastase-sensitive, consistent with intracellular glycogen. Mitotic activity was minimal in all cases. Perineural and vascular invasion were identified in 66% (2/3) of cases.

Based on the histopathological features described, it is evident that the distinction between HCCC and CCOC cannot rely solely on histopathological findings in H&E-stained sections. While salivary (soft tissue) origin (HCCC) vs. intraosseous occurrence (CCOC) was identified as the principal distinguishing feature in localized tumours, in anatomically ambiguous palate/maxilla, subtle distinguishing features such as dense stromal hyalinization (4/5), glandular differentiation (2/5), the presence of a few mucin-containing cells (4/5) and close association to salivary tissue (5/5) favoured a diagnosis of HCCC, while focal stromal hyalinization (3/3), larger tumour lobules (3/3) and peripheral palisading (2/3) favoured a diagnosis of CCOC. Immunohistochemistry offers limited additional value in this context. Cytokeratin 19 (CK19), previously considered a marker of odontogenic origin, is now known to be expressed in salivary gland tumours, including HCCC, and therefore cannot be used reliably to differentiate between these two entities (22).

### 3.2. Literature Review

Table 2 shows the clinicopathological comparisons between 58 HCCCs and 45 CCOCs published in literature. Both tumours predominantly occurred in middle-aged to older adults, with the majority of patients falling within the 41–60 and >60-year age groups. The mean age at diagnosis was comparable between the two groups (56.33 years for HCCC and 54.11 years for CCOC), with overlapping age ranges. With respect to sex as well, both tumours showed a clear female predominance, with similar proportions of males and females in each group (1:2). There was a clear difference in site distribution between HCCC and CCOC. HCCCs predominantly involved minor salivary glands (79.31%), with a smaller proportion arising in major salivary glands (20.69%). The palate and tongue/floor of the mouth minor salivary glands were the most common locations of origin (22/46 and 18/46, respectively). In contrast, CCOC occurred within the jaws, showing a marked predilection for the mandible (71.11%) compared with the maxilla (28.89%). Among jaw lesions, tumour distribution was variable, with the highest proportion involving multiple regions. Lesions extending across the entire jaw (anterior, middle and posterior) were the most common (11/38, 28.95%). This was followed by isolated posterior involvement (8/38, 21.05%) and anterior involvement (7/38, 18.42%). Overall, these findings indicate a tendency for lesions to present with multi-regional involvement rather than being confined to a single anatomical segment within jaws. Most cases in both groups presented within 1–6 months from the time patients first noticed symptoms to seeking treatment (HCCC: 53.57%; CCOC: 68.2%), followed by durations of >6 months. A small proportion of HCCCs presented within <1 month (7.14%), while none were reported for CCOCs. However, duration data were unavailable for a substantial number of cases, and the reliability of the reported durations may be limited as they are based on patient recall. In both groups, the majority of tumours measured >2 cm, with a slightly higher proportion of larger lesions (>4 cm) observed in CCOCs (44.83%) compared with HCCCs (37.25%). Surgery alone was the predominant treatment in both groups (HCCC: 76.47%; CCOC: 77.27%), while a smaller proportion received surgery with adjuvant therapy (HCCC: 23.53%; CCOC: 22.72%). One CCOC patient died before treatment could be initiated. Overall, treatment patterns were comparable between the two tumour types. In HCCCs, 91.86% of patients were alive at follow-up (74.42% disease-free and 18.60% with disease), while 6.98% had died due to disease. Similarly, in CCOCs, 88.89% were alive (80.56% disease-free and 8.33% with disease), and 11.11% had died due to disease.

Overall, no striking differences between HCCC and CCOC across most clinicopathological parameters evaluated, including age at diagnosis, sex distribution, tumour size, treatment modality and clinical outcome, were observed (Table 2). A slight difference was observed only in the anatomical site of occurrence, with soft tissue salivary origin reflected in HCCCs and intraosseous location for CCOCs. Collectively, these findings suggest broadly comparable clinical behaviour between HCCC and CCOC, with site of origin being the primary distinguishing feature.

### 3.3. Combined Case Series and Literature Review-Based Analysis of HCCCs Arising in Palatal Minor Salivary Glands and Maxillary CCOCs

Table 3 shows the clinicopathological presentations of 25 palatal HCCCs and 14 maxillary CCOCs, specifically focusing on lesions arising in palatal minor salivary glands and maxilla in contrast to the broader overall distributions described in Table 2. Both HCCCs and CCOCs in these anatomically specific sites showed a similar age distribution, with most cases occurring in the 41–60-year group and comparable mean ages (48.68 vs. 49.54 years). A female predominance was observed in both groups, with broadly similar sex distribution patterns. With respect to site, HCCC showed a strong palatal predilection with majority being confined to the palate (80%) and occasional multi-regional extension (20%), whereas CCOC demonstrated an intraosseous maxillary distribution with involvement of multiple jaw regions (42.86%), including palatal involvement (28.57%), in a minority of cases. Therefore, for extensive lesions involving multiple regions, the true site of origin may be difficult to determine, limiting reliance on distribution alone for differentiation.

Both tumour types showed a similar pattern in symptom duration, with most cases presenting after more than 6 months. HCCCs tended to present as smaller lesions compared with CCOCs, with a higher proportion of cases being ≤2 cm in size, whereas CCOCs more frequently exceeded 2 cm. Treatment patterns were comparable, with surgery alone being the main modality in both groups and a smaller proportion receiving adjuvant therapy. Metastasis was rare in both tumours, with no cases in HCCC and only occasional cases in CCOC. When outcomes were grouped as alive versus death, both tumours showed predominantly survival; however, HCCC had no disease-related deaths but more patients alive with disease, while CCOC showed more patients alive without disease with a small proportion of deaths.

No differences were observed between palatal HCCC and maxillary CCOC across all parameters. However, in contrast to HCCC, CCOC demonstrated longer symptom duration, larger lesion size, and slightly worse overall outcome. Overall, these findings indicate considerable clinicopathological overlap between palatal HCCC and maxillary CCOC, despite moderate trends in symptom duration, lesion size and outcome.

Across the literature-based overall sample (Table 2) and the palatal/maxillary series (Table 3), HCCC and CCOC showed broadly comparable clinicopathological features, including similar age distribution, female predominance, symptom duration, treatment approach and overall outcomes. Lesion size showed some variation in the datasets, with HCCC tending to present as smaller lesions, while CCOC more often exceeded 2 cm, especially in the maxillary/palatal lesions. Metastasis remained uncommon in both groups. Accordingly, HCCCs without bone invasion can be distinguished based on anatomical distribution; however, in cases with extensive multi-regional involvement, the true site of origin may be difficult to determine, limiting the diagnostic value of distribution alone.

## 4. Discussion

According to the present case series and the literature review, HCCC and CCOC are both predominantly diseases that occur in adults with a female predilection. These findings are similar to findings observed by Yang et al. [3] analysing 155 and Albergotti et al. [7] analysing 130 HCCC. Similarly, Labrador et al. [11] and Guastaldi et al. [12] analysing 117 and 107 CCOCs reported comparable demographic patterns. With respect to site of occurrence, the present findings are also in agreement with the published literature on HCCC, which demonstrates a marked predilection for minor salivary glands over major salivary glands [3,7]. However, unlike previous studies by Yang et al. [3] and Albergotti et al. [7], which included HCCCs arising in a broader head and neck anatomical spectrum, including the oropharynx, larynx, nasal cavity and nasopharynx, the present study specifically focused on lesions arising within the oral cavity (minor salivary glands) and major salivary glands. This more restricted anatomical inclusion in the present study therefore provides a more focused assessment of intraoral minor salivary gland and major salivary gland-based HCCC distribution. Similarly, studies on CCOC by Labrador et al. [11] and Guastaldi et al. [12] also demonstrate a predilection for the mandible compared with maxilla. Therefore, demographic profile with respect to age at diagnosis, sex and site distribution observed in the present case series/literature review are broadly consistent with those published in the literature for both HCCC [3,7] and CCOC [11,12].

Regarding the duration of symptoms prior to treatment, HCCC generally demonstrated shorter delays compared with CCOC, most likely due to greater visibility and accessibility of salivary gland lesions. In contrast, palatal and maxillary lesions irrespective of the tumour type were associated with a longer delay prior to presentation (Table 2 and Table 3). This may be explained by site-specific anatomical and clinical factors. Palatal lesions are often not readily visible to patients and may remain asymptomatic or minimally symptomatic for prolonged periods, leading to delayed recognition. In addition, maxillary lesions may spread within the medullary and porous cancellous bone with relatively limited early cortical expansion, allowing substantial intraosseous extension before becoming clinically evident. Consequently, by the time these lesions produce visible swelling or symptoms, they may already be relatively advanced, contributing to adverse outcomes. A higher proportion of larger maxillary CCOCs at diagnosis compared with HCCCs in the present series (Table 3) may therefore reflect their intraosseous growth pattern and delayed clinical detection rather than indicating faster tumour growth and more aggressive biological behaviour.

The considerable clinicopathological overlap between HCCC and CCOC may create diagnostic challenges in anatomically complex palatal/maxillary lesions. Accurate assessment of intraosseous involvement through radiological correlation and intraoperative evaluation remains important, particularly in lesions with possible maxillary bone involvement, as the extent of osseous spread may influence surgical planning and marginal clearance. Negative surgical margins remain an important treatment objective for both tumours, while adjuvant radiotherapy has been reported in selected cases with positive margins, perineural invasion, locally advanced disease and nodal metastasis [3,7,12]. Furthermore, subtle histopathological differences may not always be appreciable on limited biopsy or frozen section material, highlighting the importance of multidisciplinary clinicopathological correlation during surgical decision-making.

In terms of biological behaviour, both the present findings and the literature consistently demonstrate that HCCC and CCOC are low-grade malignant tumours with limited but definite metastatic potential. Yang et al. [3] and Albergotti et al. [7] reported nodal and occasional distant metastases in HCCC, while the present series shows metastasis in only a small proportion of cases, with CCOC demonstrating a relatively higher but still limited metastatic rate. This finding could be partially attributed to anatomical factors such as intraosseous growth, cohort heterogeneity, publication bias and diagnostic overlap between these two entities, rather than definite biological divergence. Similarly, recurrence is an important but infrequent event in both entities. Published series by Yang et al., Albergotti et al., Labrador et al. and Guastaldi et al. [3,7,11,12] report local recurrence in a minority of cases, a pattern that is mirrored in the present study. Nevertheless, despite their generally indolent behaviour with favourable prognosis, both tumours retain a definite potential for recurrence and metastasis, emphasizing the importance of long-term follow-up.

The histopathological diagnostic challenges encountered in the present case series when differentiating HCCC from CCOC in anatomically ambiguous palate and maxilla were addressed by identifying subtle histopathological clues. Diffuse dense hyalinization, focal glandular differentiation, mucin-containing cells and salivary gland association favoured the diagnosis of HCCC, whereas patchy hyalinization, larger tumour lobules and peripheral palisading favoured the diagnosis of CCOC. Although focal mucin-containing cells traditionally favour HCCC, Bishop et al. [85] reported that mucin-containing cells may occasionally also occur in CCOC. Similarly, peripheral palisading with reverse nuclear polarity, a feature suggestive of CCOC, has also been described in HCCC [22,85]. The use of CK19 as an odontogenic marker is likewise not entirely specific. In addition, reports of intraosseous (maxillary and mandibular) HCCC further obscure the distinction between these entities [3,7]. Collectively, these findings indicate that no single histopathological feature is diagnostic, and distinction between the two entities relies on a composite assessment of radiological, anatomical and histopathological features rather than any single criterion.

The marked clinicopathological overlap between HCCC and CCOC raises the possibility that these entities may be closely related within a single biological spectrum [1,85]. This concept is further strengthened by their shared molecular alteration, namely the EWSR1 gene rearrangement [1,9,11,21,23,60,67,72,85], which has been demonstrated in both HCCC and CCOC, supporting a potential common pathogenetic pathway. In this context, CCOC may represent the intraosseous counterpart of HCCC, similar to the established relationship between the salivary and intraosseous (central) variant of mucoepidermoid carcinoma [1,85], which share overlapping histopathological features and a molecular profile of MAML2 translocations but differ primarily in anatomic location. The fifth fascicle of Salivary Tumour Pathology also recognizes CCOC as analogous to HCCC [85], further strengthening our observation. However, although the current WHO Head and Neck Tumour classification continues to recognize HCCC and CCOC as distinct entities, substantial overlap in morphology, immunophenotype, molecular alterations and biological behaviour suggest that their relationship warrants further clarifications with respect to nomenclature in future revisions of WHO Head and Neck Tumour pathology. The present findings therefore support the concept that these lesions may represent site-related variants within a closely related biological spectrum rather than entirely unrelated lesions.

Distinguishing HCCC from other primary salivary gland tumours with clear cell morphology, particularly clear cell variants of mucoepidermoid carcinoma (MEC) and acinic cell carcinoma (AciCC), is essential due to significant diagnostic overlap. Clear cell MEC may closely resemble HCCC when mucous cells are inconspicuous; however, careful histological examination typically reveals a mixture of epidermoid, intermediate, and mucous cells, often with at least focal evidence of mucous differentiation, in MEC. In contrast, HCCC is composed of relatively monomorphic clear to eosinophilic cells arranged in cords, nests and trabeculae within a characteristic densely hyalinized stroma, lacking the cellular heterogeneity seen in MEC. Immunohistochemical analysis is not useful to distinguish the two tumours as both demonstrate HMWK and p63 while presenting as negative for s-100 and myoepithelial markers [22,85]. Similarly, AciCC, particularly its clear cell-rich variants, may mimic HCCC due to cytoplasmic clearing; however, AciCC typically shows serous acinar differentiation with basophilic granular cytoplasm and identifiable zymogen granules in at least a subset of tumour cells, along with a wider spectrum of architectural patterns including microcystic, papillary-cystic or solid growth. In contrast, HCCC demonstrates a more uniform cytomorphology and a consistently hyalinized stromal background without serous acinar differentiation. Immunohistochemically, both entities may express cytokeratin, but AciCC often shows DOG1 and amylase positivity, whereas HCCC is characteristically negative for acinar markers and frequently harbours EWSR1 gene rearrangement, supporting its distinct molecular identity. Therefore, careful attention to architectural uniformity, stromal characteristics and appropriate immunohistochemical and molecular profiling is critical in distinguishing HCCC from its histological mimics, particularly clear cell MEC and acinic cell carcinoma. It is also important to exclude metastatic clear cell RCC, which can closely mimic HCCC due to its clear cytoplasm and nested architecture. However, RCC typically lacks the characteristic hyalinised stromal background of HCCC and shows a rich sinusoidal vascular network. Immunohistochemically, HCCC shows diffuse positivity for cytokeratins and EMA with frequent EWSR1 gene rearrangement, whereas RCC is characteristically positive for PAX8, CD10 and RCC marker and negative for EWSR1 rearrangement. Thus, the integration of morphology, immune profile, and molecular findings is essential to distinguish HCCC from other primary salivary gland clear cell tumours and metastatic RCC, particularly in minor salivary gland sites of the oral cavity where diagnostic ambiguity is highest [22,85].

The histopathological differential diagnosis of CCOC includes clear cell-rich ameloblastoma, which may closely simulate CCOC; however, ameloblastoma typically demonstrates characteristic peripheral palisading of columnar cells with reverse nuclear polarity and central stellate reticulum-like areas, features that are absent in CCOC, where the tumour is composed of uniform sheets, nests, and cords of clear cells with an infiltrative growth pattern. Similarly, clear cell variants of calcifying epithelial odontogenic tumour (CEOT) may enter the differential diagnosis, but CEOT is distinguished by the presence of extracellular amyloid-like material and characteristic Liesegang ring calcifications, which are not seen in CCOC [1].

The present study has a few limitations. The number of cases included was relatively small compared with larger published series and systematic reviews, mainly due to limited access to literature, which may affect the strength of some comparisons. In addition, the literature review was mainly based on PubMed indexed studies, so relevant studies indexed in other databases may not have been included. In addition, potential sources of bias included publication bias toward unusual or aggressive cases, variable diagnostic criteria that changed over the years and inconsistent follow-up documentation. Furthermore, given the rarity of both tumours and limited subgroup sizes, statistical analyses were primarily exploratory in nature. Despite this, the study provides a focused and clinically relevant comparison of HCCC and CCOC within anatomically relevant oral and maxillofacial sites. By restricting analysis to intraoral and salivary gland lesions, it offers a more uniform assessment of key parameters such as site, size, duration, metastasis and outcomes. It also integrates institutional findings with published literature to better highlight the overlapping clinicopathological and molecular features of these entities, including their shared EWSR1 gene rearrangement (based on literature), supporting their close biological relationship.

## 5. Conclusions

HCCC and CCOC demonstrate considerable clinicopathological and molecular overlap, including occurrence in adults, female predilection, generally indolent clinical behaviour and shared EWSR1 gene rearrangement, supporting a close biological relationship between the two entities. Although CCOC may demonstrate relatively larger lesions and higher metastatic frequency, these differences may partly reflect anatomical factors related to intraosseous growth rather than definite biological divergence. The overlap between these tumours, particularly palatal and maxillary lesions, support the hypothesis that CCOC may represent the intraosseous counterpart of HCCC and highlight the importance of integrated clinicopathological assessment and further clarification in future WHO classifications.

## Figures and Tables

**Table 1:** (**A**): Demographic presentations of HCCC and CCOC included in the case series. (**B**): Histopathological presentations of HCCC and CCOC included in the case series.

**(A)**															
	**Age**		**Sex**	**Site**			**Size**		**Treatment**		**Recurrence**		**Follow-Up/** **Outcome**		
**HCCC**															
Case 1	62		F	Maxilla/ Palate			10 cm		Surgery/ Radiotherapy		Absent		5 months/Alive		
Case 2	58		M	Palate			7 cm		Surgery		Present (192 months)		195 months/Alive		
Case 3	22		F	Floor of the mouth			4 cm		Surgery		Not available		Not available		
Case 4	56		F	Palate			>4 cm		Surgery		Not available		Not available		
Case 5	56		M	Buccal mucosa			5 cm		Surgery		Absent		24 months/Alive		
**CCOC**															
Case 1	58		F	Mandible			>4 cm		Surgery		Present (48 months)		60 months/Alive		
Case 2	74		M	Mandible			2 cm		Surgery		Absent		24 months/Alive		
Case 3	51		F	Maxilla			3 cm		Surgery		Present (72 months)		96 months/Alive		
**(B)**															
		**HCCC** **Case 1**			**HCCC Case 2**	**HCCC Case 3**		**HCCC Case 4**		**HCCC Case 5**		**CCOC Case 1**		**CCOC Case 2**	**CCOC** **Case 3**
**Encapsulation**		Invasive			Invasive	Invasive		Invasive		Invasive		Invasive		Invasive	Invasive
**Morphological presentation** Thin strands Large islands Peripheral palisading Glandular change		Present Present Absent Present			Present Absent Present Present	Present Present Absent Absent		Present Absent Absent Absent		Present Present Absent Absent		Present Present Present Absent		Absent Present Absent Absent	Present Present Present Absent
**Cell types** Clear cells Cells with eosinophilic cytoplasm		+++ ++			+++ ++	+++ ++		+++ ++		+++ Absent		+++ +		+++ +	+++ Absent
**Stroma** Hyalinization		Dense and diffuse			Dense and diffuse	Focally present		Dense		Dense and diffuse		Focal		Focal	Absent
PNI		Present			Present	Absent		Absent		Absent		Present		Absent	Present
VI		Absent			Absent	Present		Absent		Present		Present		Absent	Present
Mitosis		2 per 10 HPFs			3 per 10 HPFs	1 per 10 HPFs		None		6 per 10 HPFs		2 per 10 HPFs		3 per 10 HPFs	3 per 10 HPFs
Soft tissue invasion/bone		Present Antrum /nose			Present Antrum	Absent		Absent		Extends to retromolar region		Present		Absent	Present Antral/nasal invasion
Marginal status		Involved margins			Involved margins	Involved margins		Involved margins		Involved margins		Involved margins		Clear	Involved margins
Nodal metastasis		Absent			Absent	Present		Absent		Present		Present		Absent	Absent
Distant metastasis		N/A			Absent	Absent		Absent		N/A		Absent		Present (scalp)	N/A
PAS DPAS		Positive Positive			Positive Negative	Positive Positive		Positive Positive		Positive Positive		Positive Negative		Positive Negative	Positive Negative
CK 19		Not done			Focal positive	Not done		Not done		Not done		Positive		Not done	Not done

F = Female; M = Male. PNI = Perineural invasion, VI = vascular invasion, HPFs = high-power fields, PAS = periodic acid–Schiff stain, DPAS = diastase-resistant periodic acid–Schiff stain, CK = cytokeratin, N/A = not available.

**Table 2:** Clinicopathological comparison of HCCCs and CCOCs included in the case series and literature.

Feature	HCCC *N* = 58 (%)	CCOC *N* = 45 (%)
Age at diagnosis in years		
<20	0 (0)	0 (0)
21–40	11 (18.97)	8 (17.78)
41–60	25 (43.10)	21 (46.67)
>60	22 (37.93)	16 (35.56)
Mean age	56.33	54.11
Range	30–88	25–84
Sex		
Male	18 (31.03)	14 (31.11)
Female	40 (68.97)	31 (68.89)
Site		
Major salivary glands	12 (20.69)	
Minor salivary glands	46 (79.31)	
Mandible		32 (71.11)
Maxilla		13 (28.89)
Duration		
<1 month	2 (7.14)	0 (0)
1–6 months	15 (53.57)	15 (68.2)
>6 months	11 (39.29)	7 (31.8)
Size in cm		
≤2	12 (23.52)	4 (13.79)
>2≤ 4	20 (39.22)	12 (41.38)
>4	19 (37.25)	13 (44.83)
Treatment		
Surgery only	39 (76.47)	34 (77.27)
Surgery with adjuvant therapy	12 (23.53)	10 (22.72)
Died before Rx		1
Metastasis		
Present	3 (6.97)	7 (17.5)
Absent	40 (94.03)	33 (82.5)
Outcome (mean follow-up period in months)	38.41	32.03
Alive without disease	32 (74.42)	29 (80.56)
Alive with disease	8 (18.60)	3 (8.33)
Dead due to disease	3 (6.98)	4 (11.11)

**Table 3:** Clinicopathological comparison of HCCCs of minor salivary glands of the palate and CCOCs of the maxilla.

Feature	HCCC *N* = 25 (%)	CCOC *N* = 14 (%)
Age at diagnosis in years		
<20	0 (0.00)	0 (0.00)
21–40	8 (32.00)	4 (28.57)
41–60	12 (48.00)	6 (42.86)
>60	5 (20.00)	4 (28.57)
Mean age	48.68	49.54 yrs
Range	22–85	26–68
Sex		
Male	9 (36.00)	6 (42.86)
Female	16 (64.00)	8 (57.14)
Location within jaw lesions		
Anterior (3–3)	0 (0.00)	0 (0.00)
Posterior (6–8)	0 (0.00)	4 (28.57)
Middle (4–5)	0 (0.00)	2 (14.25)
A + M	1 (4.00)	1 (7.10)
M + P	0 (0.00)	2 (14.25)
A + M + P	4 (16.00)	2 (14.25)
Palate	20 (80.00)	1 (7.10)
Duration		
<1 month	1 (7.10)	0 (0.00)
1–6 months	5 (35.71)	5 (57.14)
>6 months	8 (57.14)	3 (42.86)
Size in cm		
≤ 2	8 (34.78)	0 (0.00)
>2≤ 4	7 (30.44)	5 (62.5)
>4	8 (34.78)	3 (37.5)
Treatment		
Surgery only	16 (76.2)	10 (71.43)
Surgery with adjuvant therapy	5 (23.8)	4 (28.57)
Metastasis		
Present	0 (0.00)	1 (10.00)
Absent	16 (100.00)	9 (90.00)

## Data Availability

No new data were created or analyzed in this study.

## Abbreviations

The following abbreviations are used in this manuscript:

HCCC	Hyalinizing clear cell carcinoma
CCOC	Clear cell odontogenic carcinoma
DPAS/PAS	Diastase-resistant periodic acid–Schiff stain/periodic acid–Schiff stain
EWSR1	Ewing sarcoma breakpoint region 1
